# Rab3Gap1 mediates exocytosis of Claudin-1 and tight junction formation during epidermal barrier acquisition

**DOI:** 10.1016/j.ydbio.2013.04.034

**Published:** 2013-08-15

**Authors:** G. Youssef, L. Gerner, A.S. Naeem, O. Ralph, M. Ono, C.A. O’Neill, R.F.L. O’Shaughnessy

**Affiliations:** a Livingstone Skin Research Centre for Children, UCL Institute of Child Health, London WC1N 1EH, UK; b Immunobiology, UCL Institute of Child Health, London WC1N 1EH, UK; c Prostate Cancer Research Group, Centre for Molecular Medicine Norway, Nordic EMBL Partnership, University of Oslo and Oslo University Hospital, N-0318 Oslo, Norway; d Dermatological Sciences Research Group, Institute of Inflammation and Repair, The University of Manchester, Manchester Academic Health Sciences Centre, Manchester M13 9PT, UK

**Keywords:** Rab3Gap1, Epidermis, Epidermal barrier, Signalling, Keratinocyte

## Abstract

Epidermal barrier acquisition during late murine gestation is accompanied by an increase in Akt kinase activity and cJun dephosphorlyation. The latter is directed by the Ppp2r2a regulatory subunit of the Pp2a phosphatase. This was accompanied by a change of Claudin-1 localisation to the cell surface and interaction between Occludin and Claudin-1 which are thought to be required for tight junction formation.

The aim of this study was to determine the nature of the barrier defect caused by the loss of AKT/Ppp2r2a function. There was a paracellular barrier defect in rat epidermal keratinocytes expressing a Ppp2r2a siRNA. In Ppp2r2a knockdown cells, Claudin-1 was located to the cytoplasm and its expression was increased. Inhibiting cJun phosphorylation restored barrier function and plasma membrane localisation of Claudin-1. Expression of the Rab3 GTPase activating protein, Rab3Gap1, was restored in Ppp2r2a siRNA cells when cJun phosphorylation was inhibited. During normal mouse epidermal development, Claudin-1 plasma membrane localisation and Rab3Gap1 cell surface expression were co-incident with Akt activation in mouse epidermis, strongly suggesting a role of Rab3Gap1 in epidermal barrier acquisition. Supporting this hypothesis, siRNA knockdown of Rab3Gap1 prevented plasma membrane Claudin-1 expression and the formation of a barrier competent epithelium. Replacing Rab3Gap1 in Ppp2r2a knockdown cells was sufficient to rescue Claudin-1 transport to the cell surface. Therefore these data suggest Rab3Gap1 mediated exocytosis of Claudin-1 is an important component of epidermal barrier acquisition during epidermal development.

## Highlights

• Barrier acquisition correlates with Ppp2r2a and cell surface Claudin-1 expression.
• Ppp2r2a knockdown results in a paracellular barrier defect.
• Ppp2r2a knockdown prevents cell-surface claudin-1 expression in a c-Jun dependent fashion.
• Barrier rescue by inhibition of c-Jun phosphorylation involves exocytosis and Rab3Gap1.
• Rab3Gap1 is induced during barrier acquisition and is necessary for cell surface claudin-1.

## Graphical abstract

**Figure:**
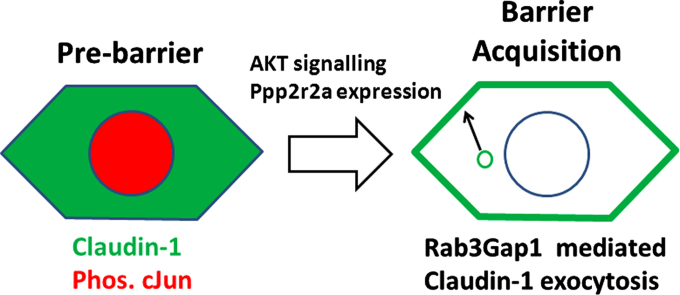

## Introduction

A principle cause of mortality and morbidity in severely premature births is the inability of epithelia such as the epidermis to be barriers to the external environment. A better understanding of the mechanisms of epidermal barrier acquisition could lead to novel therapies (Smith et al., 2010; Kalia et al., 1998; Sharma et al., 2007); however, this process is difficult to investigate in the human foetus. Murine epidermal barrier acquisition occurs in the same way as in humans: a “wave” of barrier competence passes across the epidermis and converges at the dorsal and ventral midlines (Hardman et al., 1998). Within 24 h, the epidermis becomes impermeable to small solutes and forms a barrier to dye penetration (Hardman et al., 1998; Byrne et al., 2010). The serine–threonine kinase Akt is activated coincident with barrier acquisition and Akt inhibition prevents barrier acquisition (O'Shaughnessy et al., 2009). cJun dephosphorylation occurs transiently, in an Akt-dependent manner, during epidermal barrier acquisition (O'Shaughnessy et al., 2009). When cJun dephosphorylation is prevented in skin explants, barrier acquisition is abolished. Furthermore, siRNA knockdown of Ppp2r2a, a regulatory subunit of the Pp2a phosphatase which targets cJun for dephosphorylation, delays barrier acquisition in organotypic cultures (O'Shaughnessy et al., 2009). Jun Kinase inhibition restores barrier function in these cultures highlighting the critical role of cJun dephosphorylation by Ppp2r2a in epidermal barrier function. To date the downstream targets of cJun phosphorylation during epidermal barrier acquisition are not known.

Although epidermal barrier function was previously thought to be provided solely by the stratum corneum, more recent work has demonstrated that tight junction complexes are critical components of the epidermal barrier. Tight junctions in simple epithelia such as the lung and gut provide a permeability barrier preventing paracellular diffusion of solutes and ions (Tsukita and Furuse, 2002). Tight junctions are composed of the obligate component Occludin (Furuse et al., 1993) and proteins known as Claudins (Furuse and Tsukita, 2006). The importance of Claudin-1 is demonstrated in knockout mice which die from epidermal dehydration due to severely impaired barrier function (Furuse et al., 2002). Although Occludin null mice have no gross skin barrier defect (Saitou et al., 2000), Occludin knockdown cells, including keratinocytes, exhibit reduced paracellular barrier function (Cong et al., 2013; Kirschner et al., 2013). Claudin-1 is expressed prior to epidermal barrier acquisition (Troy et al., 2007; Lopardo et al., 2008), while Occludin is expressed in the uppermost granular layer just prior to birth (Morita et al., 1998). Although both are expressed during barrier acquisition, the relationship between Claudin-1 and Occludin expression and localisation and barrier acquisition in epidermis has not yet been addressed.

The transport of tight junction components to the cell surface involves the coordinate regulation of Rab GTPases, with various Rabs being implicated in the transport of apical junctions in epithelia. Only 2 of the 60 Rabs can be detected in the vicinity of tight junctions in epithelia (Marzesco and Zahraoui, 2005; Weber et al., 1994). In particular Rabs 3 and 13 are required for the dynamic endocytic recycling of Occludin and Claudin-1 to the cell surface (Morimoto et al., 2005; Yamamura et al., 2008; Yamamoto et al., 2003). Save for a role for Rab3A in melanosome transport in melanocytes (Scott and Zhao, 2001), nothing is known about the expression and function of these Rabs in epidermal barrier acquisition.

In this study, we show that the cell surface localisation of Claudin-1 and association with Occludin occur during epidermal development and that this is concomitant with Akt activation and Ppp2r2a expression. siRNA knockdown of Ppp2r2a causes paracellular barrier defects associated with the cytoplasmic accumulation of Claudin-1. Jun Kinase inhibition rescues paracellular barrier function by restoring Claudin-1 plasma membrane expression. We provide evidence that Rab3-mediated exocytosis of Claudin-1 is concomitant with this barrier rescue, and that the GTPase protein Rab3Gap1 is necessary for cell surface Claudin-1 and Occludin Expression.

## Materials and methods

### Immunofluoresence and microscopy

Immunofluoresence was performed using standard techniques. Briefly, keratinocytes were fixed for 10 min at 4 °C in 4% paraformaldehyde in phosphate buffered saline (PBS), then permeabilised in 0.2% Triton X-100 (Sigma, Gillingham, UK) for 30 min. Following this samples were incubated with the primary antibodies. These were: rabbit anti pSer473 Akt 1/10; rabbit anti pSer63 cJun 1/10 (both Cell Signalling Technologies, Danvers, USA); rabbit anti Claudin-1 (Invitrogen, Paisley, UK) 1/25; mouse anti Occludin (Invitrogen, Paisley, UK) 1/25; rabbit anti PR55alpha (Santa Cruz Biotechnologies, Santa Cruz, US) 1/25; rabbit anti Zonula Occludins-1 (Santa Cruz Biotechnologies, Santa Cruz, US) 1/50; Mouse anti Rab3 (BD Biosciences, Oxford, UK) 1/25. Rabbit anti Rab3Gap1 (Abgent, Cambridge, UK) 1/25. Antibody detection was performed using either the Elite Avidin–Biotin-Complex system and DAB (Vector Laboratories, Burlingame, US) or Alexa 488 (green) and 536 (red) conjugated secondary antibodies (Molecular Probes, Invitrogen, Paisley, UK ) at 1/800. Cells and Organotypic cultures were counterstained using haemotoxilin for immunohistochemistry and 4′,6-diamidino-2-phenylindole (DAPI) for immunofluoresence. Images were taken with a Nikon Eclipse E600 microscope with a Coolsnap digital camera (MediaCybernetics, Bethesda, MD,USA) and ImagePro 6.0 software (MediaCybernetics), or a Zeiss laser scanning confocal microscope. Image analysis was performed using ImageJ (http://rsb.info.nih.gov/ij/).

### Proximity ligation analysis

Proximity ligation analysis (PLA assay, OLink Bioscience, Uppsala, Sweden; (Soderberg et al., 2006)), determines *in situ* whether 2 proteins are within 38 nm of each other, and potentially interact. PLA was performed according to manufacturer's protocols on frozen embryonic mouse sections and REKs fixed in 4% paraformaldehyde and 0.2% Triton-X 100. Occludin and Claudin-1 or Zo-1 and Occludin primary antibodies were simultaneously incubated at 1/25 concentrations. Subsequently sections or cells were incubated for 2 h at 37 °C with a 1:5 dilution of the secondary antibodies conjugated with complementary DNA, anti-mouse+ and anti-rabbit−(598 nm excitation). DNA strands were hybridised with a specific probe, DNA was synthesised by rolling circle amplification and visualised by fluorescent oligonucleotides that hybridise to the synthesised DNA, with a red dot by fluorescence microscopy indicating a positive result. PLA was analysed by using the spot finding algorithms in the ImageJ software suite.

### siRNA constructs, rat epidermal keratinocyte culture and organotypic culture

SureSilencing shRNA plasmids (Qiagen, Paisley, UK) to rat Ppp2r2a (shRNA2-TGACTGGATCCTACAATAATT) (O'Shaughnessy et al., 2009) and Rab3Gap1 (shRNA1-ATAGCCCATTCCAGCAAAGTT, shRNA4-CGACAGTCTAACATACAAACT) were transfected into rat epidermal keratinocytes (REKs) using lipofectamine plus (Invitrogen, Paisley, UK). A Myc tagged Rab3Gap1 expressing plasmid (OriGene, Rockville, Maryland, USA) was transiently transfected into Ppp2r2a knockdown cells using lipofectamine 2000 (Qiagen, Paisley, UK). REKs were selected when necessary in 100 μM G418 (Invitrogen, Paisley, UK) and passaged in DMEM +10% foetal calf serum. Organotypic culture was performed as described in O'Shaughnessy et al., 2007: Briefly, 2×10^5^ REKs were cultured under selection on de-epidermised dermis (Euro Skin Bank, Beverwijk, Netherlands). Confluent cultures were raised to the air–liquid interface and cultured for up to 10 days. Organotypic cultures were fixed in 4% paraformaldehyde prior to the haemotoxilin barrier assay, or embedded in OCT for cryosectioning. For visualising barrier competence, organotypic cultures were passed up and down a methanol gradient, placed in 1% haemotoxilin in water for 1 min, and embedded in paraffin.

The Jun Kinase inhibitor SP600125 (Sigma, Gillingham, UK), was added to submerged confluent cultures for 24 h at a concentration of 50 nM. Organotypic cultures were treated every 24 h with 50 nM SP600125 or vehicle (DMSO) from 5 days post-raising to the air–liquid interface. Concanamycin C (Sigma, Gillingham UK) was added to submerged confluent cultures at a concentration 2 μM for 2 h prior to further western and imunoflouresence analysis, and for up to 24 h for Transepidermal electrical resistance and FITC-dextran penetration experiments

### Analysis of TEER and FITC dextran penetration

REKs were seeded at confluent density on Thincert^™^ (Greiner) cell culture inserts (mean pore size of 0.4 uM). TEER was measured every 24 h using an Evometer (World Precision Instruments ltd, Stevenage, UK) fitted with chopstick electrodes. Monolayer resistance was normalised to the surface area of the cell culture insert and the TEER of blank filters was subtracted from the measured value of the monolayer. A total of 6 individual experiments containing 3 replicates within each experiment were performed. Measurement of FITC-dextran penetration was performed by adding FITC-dextran at a final concentration of 0.2 mg/ml to the apical side of the monolayer. Basal medium was collected after 3 h incubation at 37 °C and fluorescence measured from a standard curve using a Wallac 1420 Victor2 fluorimeter (Wallac OY, Finland). The excitation and emission wavelengths were 485 nm and 535 nm respectively. The data shown are mean±standard deviation. Data was analysed using MannWhitney U test for non-parametric data or students *T* test as appropriate.

### Western blot analyses

Protein lysates were prepared from REKs in total lysis buffer, Tris HCl pH 7.5, 5% SDS and 20% beta-mercaptoethanol, Lysates were separated on 7.5–10% SDS/polyacrylamide gels and transferred onto nitrocelluose filters (Hybond C+ – Amersham). Primary antibodies and concentrations were as follows: rabbit anti PR55a (Ppp2r2a) (Santa Cruz Biotechnologies, Santa Cruz, US) 1/500; rabbit anti cJun (Cell Signalling Technologies, Danvers, USA) 1/100; rabbit anti pSer63 cJun (Cell Signalling Technologies, Danvers, USA) 1/100; rabbit anti Claudin-1 (Invitrogen, Paisley, UK) 1/500; mouse anti Occludin (Invitrogen, Paisley, UK ) 1/500; Rabbit anti Zonula Occludins-1 (Santa Cruz Biotechnologies, Santa Cruz, US) 1/500; Mouse anti Rab3 (BD biosciences, Oxford, UK) 1/100. Rabbit anti Rab3Gap1 (Abgent, Cambridge, UK) 1/100; Rabbit anti Exoc3 (Proteintech, Chicago, Illinois, USA) 1/500; Rabbit anti Psmd4 (Cell Signalling Technologies, Danvers, USA) 1/500; Mouse anti c-Myc (Clone 9E10) (Cambridge Bioscience, Cambridge, UK) 1/500; mouse anti Gapdh (Millipore, Watford, UK) 1/1000 Primary antibody incubations were in PBS-Tween or TBST (100 mM Tris HCl, 0.2 M NaCl, 0.1% Tween-20 (v/v)) containing 5% bovine serum albumin (Sigma, Gillingham, UK) overnight at 4 °C or for 1–2 h at room temperature. Secondary antibody incubations were in 5% skimmed milk powder for 1 h at room temperature. The following concentrations were used; swine anti-rabbit HRP 1:5000; rabbit anti-mouse HRP (DakoCytomation, Cambridge, UK) 1:2000; donkey anti-goat HRP (Jackson Immunoresearch, Newmarket, UK) 1:3000. Protein was visualised using the ECL plus kit (GE Healthcare, Buckinghamshire, UK). Western blot exposures were scanned and then densitometry analysis was performed where necessary using the ImageJ suite (http://rsb.info.nih.gov/ij/).

### Microarray analysis and analysis of gene expression data

Probes were synthesised from RNA extracted from triplicate scrambled control cultures and duplicate Ppp2r2a kd cultures and kd Cultures treated for 24 h with 50 nM SP600125. This was hybridised to Affymetrix Rat Exon ST 1.0 Arrays (Affmetrix, Santa Clara, *CA*, USA). This was performed by the UCL genomics core facility. Microarray data were normalised using *mas5* of *affy*. Differentially expressed genes were identified in Ppp2r2a kd cells and kd cells treated with SP600125 by *p*<0.05 considering a false discovery rate by the Bioconductor package *limma*. Hierarchical clustering was performed according to Euclidean distance using the complete agglomeration method. Over-represented Gene Ontology (GO) groups were determined using DAVID (Database for Annotation, Visualisation and Integrated Discovery—http://david.abcc.ncifcrf.gov/). GO groups were over-represented if their fold enrichment was 2-fold or higher and the *p*-value was less than 0.05 (an EASE score of 1.3 or higher in DAVID). Interactions of selected genes were identified using STRING (Search Tool for the Retrieval of Interacting Genes/Proteins http://string-db.org).

## Results

### 1.1 Ppp2r2a is necessary for plasma membrane localisation of tight junction components and formation of the paracellular barrier

**Fig. 1:**
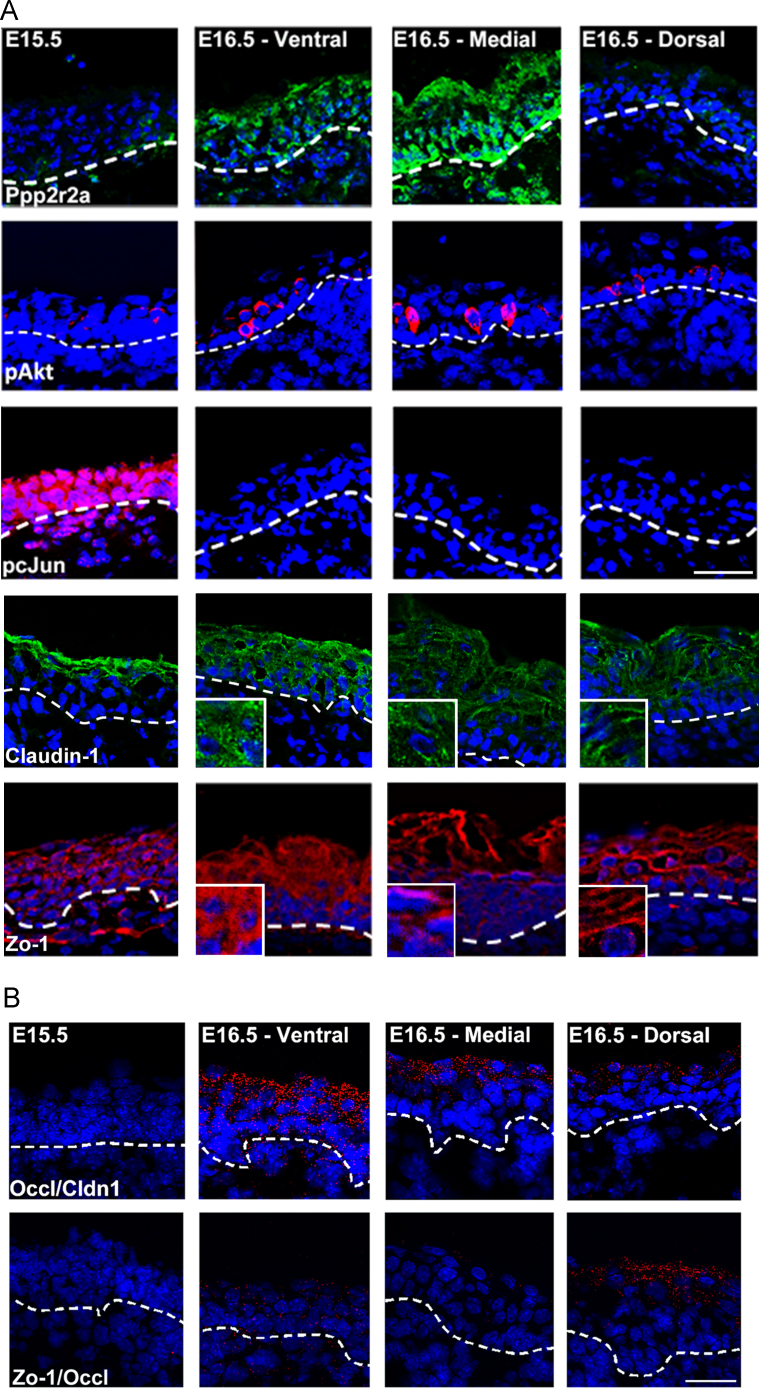
**Expression of Ppp2r2a correlates with pAKT activation, cJun dephosphorylation and interaction between Claudin-1 and Occludin during epidermal development.** (A) Representative data from immunofluorescent analysis of Ppp2r2a, pSerAkt, and pcJun, and Claudin-1 and Zo-1 of E15.5 and E16.5 embryonic epidermis from 3 different embryos. (B) Representative data from a proximity ligation experiment between Occludin and Claudin-1 and Occludin and Zo-1 antibodies in triplicate embryos. A red dot indicates that the two proteins are within 40 nm of each other, suggestive of interaction. Bar (A and B) 50 µm.

**Fig. 2:**
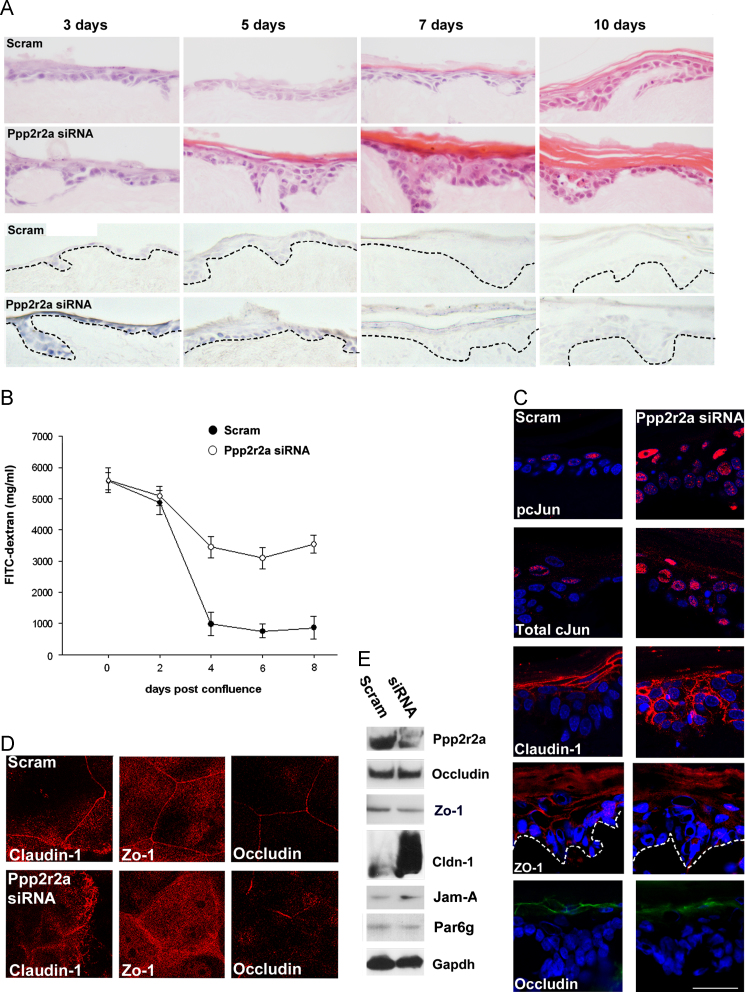
**Ppp2r2a knockdown cells have a defective paracellular barrier and altered cell surface expression of tight junction components.** (A) Haemotoxilin and Eosin stain of scrambled control and Ppp2r2a kd cultures 3,5,7 and 10 days post raising to the air–liquid interface. Lower 2 rows are the corresponding whole mount haematoxylin staining, showing delay in outside-in barrier acquisition in the Ppp2r2a kd organotypic cultures. (B) 4 kDa FITC-dextran penetration in post-confluent cells. Error bars are s.d. for triplicate results, and data is representative of *n*=6 experiments. (C) Confocal microscopy of Immunofluoresence staining of pcJun, Total cJun, Claudin-1, Zo-1 and Occludin in organotypic culture. (D) Immunofluoresence of Claudin-1, Zo-1 and Occludin in scrambled control and Ppp2r2a kd cells in post-confluent culture. (E) Western blot of Ppp2r2a, Occludin and Claudin-1, and other Tight junction components differentially expressed in Ppp2r2a kd cells, confirming Ppp2r2a knockdown and the up-regulation of Claudin-1 and Jam-A. Bar 100 µm (A), 50 µm (C), and 10 µm(D).

To begin with, we sought to clarify the relationship between Ppp2r2a expression and tight junction protein expression. In agreement with previous work we showed that Ppp2r2a expression was concomitant with known markers of epidermal barrier acquisition in the mouse such as phosphorylation of Akt and the dephosphorylation of cJun (Fig. 1A, (O'Shaughnessy et al., 2009)). Prior to barrier acquisition, both Claudin-1 and Zonula Occuldins-1 (Zo-1) expression was cytoplasmic. However post barrier acquisition both proteins were found predominantly at the cell surface, suggesting that cell surface expression is intimately associated with barrier acquisition (Fig. 1A). Proximity ligation analysis indicated a potential interaction between Claudin-1 and Occludin and between Zo-1 and Occludin during this barrier acquisition period (Fig. 1B). We therefore tested whether barrier defects correlated with tight junction defects using Ppp2r2a shRNA expressing (Ppp2r2a kd) rat epidermal keratinocytes (REK). Haemotoxilin penetration assays indicated that control organotypic cultures acquired barrier function 3 days after being raised to the air–liquid interface (Fig. 2A; (Byrne et al., 2010; O'Shaughnessy et al., 2009)). FITC-dextran permeability decreased markedly in scrambled controls 2–4 days post confluence, suggesting formation of a paracellular barrier. This was consistent with haemotoxilin penetration data in organotypic culture. 4 kDa FITC-dextran permeability was greater in the siRNA expressing cultures (Fig. 2B), suggesting that Ppp2r2a kd cells had a specific defect in their paracellular barrier. Barrier function was restored in the Ppp2r2a kd cultures concomitant with hyperkeratosis.

We then examined the expression of tight junction components in organotypic cultures (Fig. 2C). Claudin-1 was localised to the cell surface in the upper cell layers in the scrambled control organotypic cultures. In contrast, Claudin-1 expression was increased, in more cell layers in and was more cytoplasmic in Ppp2r2a kd cultures. In addition the flattened appearance of the granular layer cells was lost in the siRNA expressing cultures. Cell surface expression of Zo-1 was also lost in siRNA expressing organotypic cultures. Occludin expression was continuous in the granular layer of control organotypic cultures, while in Ppp2r2a kd cultures, Occludin expression was patchy and discontinuous (Fig. 2C). In post confluent cell culture (Fig. 2D), cell surface localisation of Claudin-1 and Zo-1 was reduced, and cytoplasmic expression was increased. Occludin expression, although still at the cell surface was discontinuous, consistent with protein expression in organotypic culture.

Gene array analysis revealed 4 up-regulated and 23 down-regulated genes related to cell–cell junctions in the Ppp2r2a kd cells compared to controls (Supplementary Fig. S1). Claudin-1 was the most up-regulated cell–cell junction gene in the Ppp2r2a kd cells (24 fold), and Jam-A was also up-regulated. Western blot confirmed the increased expression of Claudin-1 and Jam-A in the Ppp2r2a kd cells compared to scrambled control cells (Fig. 2E).

### Inhibiting cJun phosphorylation rescues tight junction protein expression and localisation and partially restores barrier function in Ppp2r2a siRNA expressing cells

**Fig. 3:**
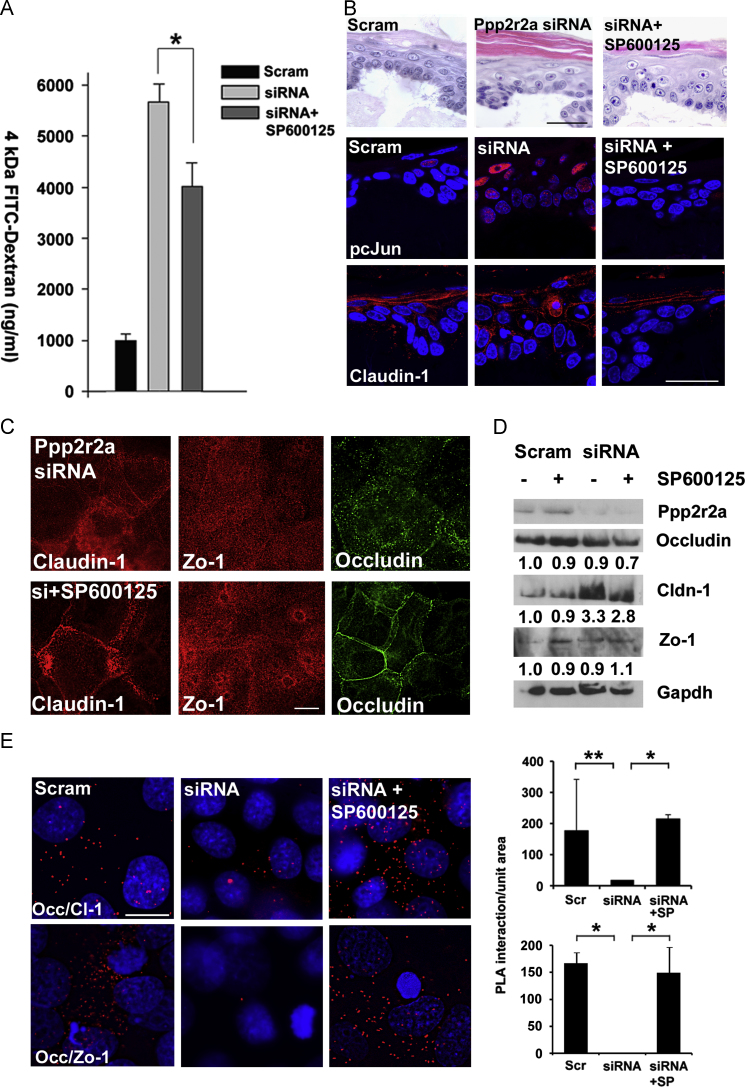
**Jun Kinase inhibition restores cell surface expression of tight junction components in Ppp2r2a siRNA expressing cells.** (A) 4 kDa FITC dextran penetration experiment at 5 days post confluence comparing scrambled controls, Ppp2r2a kd cells alone or treated with the JNK inhibitor SP600125. *, *p*-value 0.001, Two-tailed *t*-test. Error bars are s.d. (B) pcJun and Claudin-1 immunofluoresence in Scrambled controls, siRNA expressing alone or treated with SP600125, showing restoration of normal levels of pcJun and normal localisation of Claudin-1. This correlated with a reduction in hyperkeratosis in the SP600125 treated organotypics. (C) Claudin-, Zo-1 and Occludin immunofluorescence in Ppp2r2a siRNA kd cells alone or treated with SP600125. (D) Western blot analysis of Occludin, Claudin and Zo-1 in the same cells. Gapdh is the loading control. Numbers indicate the fold increase compared to scrambled controls. E. Proximity ligation analysis of Claudin-1 and Occludin and Zo-1 and Occludin interaction in Ppp2r2a kd cells and Ppp2r2a kd cells treated with the Jun Kinase inhibitor SP600125, (*p<0.05, **p<0.001, *n*=3). Bars 50 µm (B) and 10 µm (C, E).

Since previously, we showed that a JNK inhibitor SP600125, restores barrier function measured by dye penetration in Ppp2r2a kd cells in organotypic culture (O'Shaughnessy et al., 2009), we investigated the mechanism of this rescue in the context of the changes in the expression and localisation of Claudin-1, Zo-1 and Occludin. Treatment with SP600125 for 24 h in post-confluent cells resulted in a decrease in FITC-dextran permeability (Fig. 3A), suggesting that restoration of the paracellular barrier was at least partly responsible for restoration of barrier function. This treatment was also sufficient to reduce hyperkeratosis in the Ppp2r2a siRNA kd organotypic cultures (Fig. 3B).

**Fig. 4:**
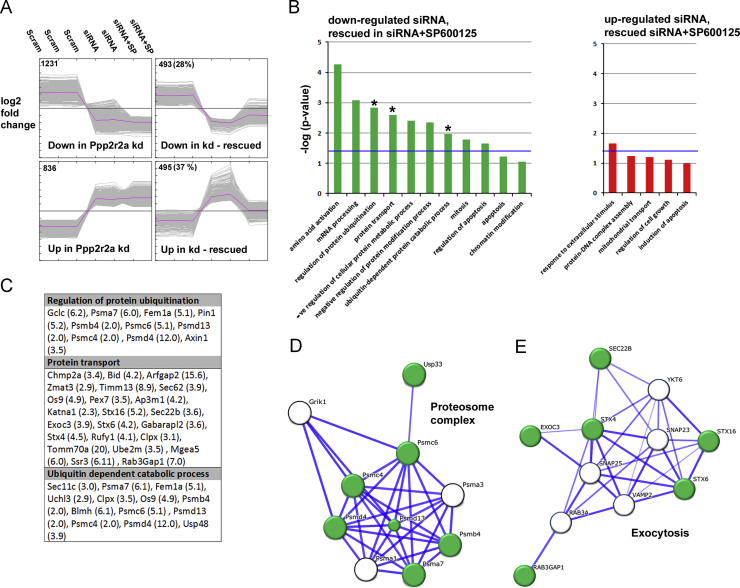
**Genes involved in exocytosis and the regulation of ubiquitin-dependent proteolysis are altered in Ppp2r2a siRNA expressing cells and restored by Jun Kinase inhibition.** (A) Clusters of differentially expressed genes were identified in the Ppp2r2a kd cells, both non-rescued and rescued by SP600125 treatment. The number in the corner is the number of differentially expressed genes in that cluster (% of all differentially expressed genes). (B) Graphical representation of over-represented gene ontology groups in differentially expressed and SP600125 rescued genes, groups with an asterisk were investigated further. The blue line denotes the *p*<0.05 significance value. (C) Table of the differentially expressed genes in each of the indicated gene ontology groups. Fold reduction in the Ppp2r2a kd cells is shown in brackets. (D and E) STRING networks of interacting down-regulated and SP600125 rescued genes involved in the proteasome complex (D) and Exocytosis (E).

Treatment of Ppp2r2a kd organotypic cultures with SP600125 restored Claudin-1 expression to the cell surface and to the upper cell layers (Fig. 3B), and restored normal granular layer cell shape to the organotypic cultures. This suggested that restoration of tight junction protein expression to the cell surface correlated with barrier restoration. In confluent keratinocytes, SP600125 treatment caused redistribution of Claudin-1 and Occludin back to the cell surface, with only a partial change of localisation in Zo-1 (Fig. 3C). Co-localisation of Claudin-1 and Occludin as the cell surface was restored by SP600125 treatment (Supplementary Fig. S2). Claudin-1 protein levels *per se* were reduced by SP600125 treatment (Fig. 3D), with no change in Zo-1 and Occludin levels observed. We used proximity ligation analysis in confluent culture and determined that the interaction between Claudin-1 and Occludin and between Zo-1 and Occludin we observed in Scrambled controls and lost in Ppp2r2a siRNA expressing cells was restored with SP600125 treatment (Fig. 4E). Therefore SP600125 treatment partially rescued epidermal barrier function by restoring cell-surface expression and interaction of tight junction components.

### Ppp2r2a knock down results in altered expression of genes involved in exocytosis which are rescued by SP600125 treatment

We hypothesised that Ppp2r2a kd cells exhibited defective cell surface transport of Claudin-1 which was corrected by SP600125 treatment. To test this we examined gene expression in scrambled control cells, Ppp2r2a kd cells and Ppp2r2s kd cells treated for 24 h with SP600125 (Fig. 4A). There were 1724 genes down-regulated and 1331 genes up-regulated by more than 1.5 fold in the Ppp2r2a kd cells (Fig. 4A). We examined the over-represented gene ontology groups in the differentially expressed genes (Supplementary Fig. S3). Genes involved in ubiquitination and protein localisation were among the down regulated genes, while genes involved in negative regulation of apoptosis were among the up-regulated genes.

493 down-regulated genes (28%) and 495 up-regulated genes (37%) were rescued by SP600125 treatment. We also examined the gene ontology groups that were over-represented in each of these clusters. Although there were no significantly over-represented gene ontology groups in the up-regulated and rescued genes, in the down-regulated, restored by SP600125 group, there were genes in involved in ubiquitination and protein transport (Fig. 4B and C). We identified networks of interacting genes specifically involved in the proteasome complex and with Exocytosis (Fig. 4D and E). We identified important proteins in these networks for further analysis. Psmd4 (S5a) is a component of the proteasome complex that binds polyubiquintinated proteins, targeting them for degradation by the proteosome (Ferrell et al., 1996), Exoc3 (Sec6), a component of the 7-protein exocyst protein complex that targets vesicles to the cell surface for exocytosis and transport of plasma membrane proteins (Inoue et al., 2003), and Rab3Gap1 a Rab3 GTPase activating protein which recycles Rab3 for further rounds of exocytosis (Sakane et al., 2006).

**Fig. 5:**
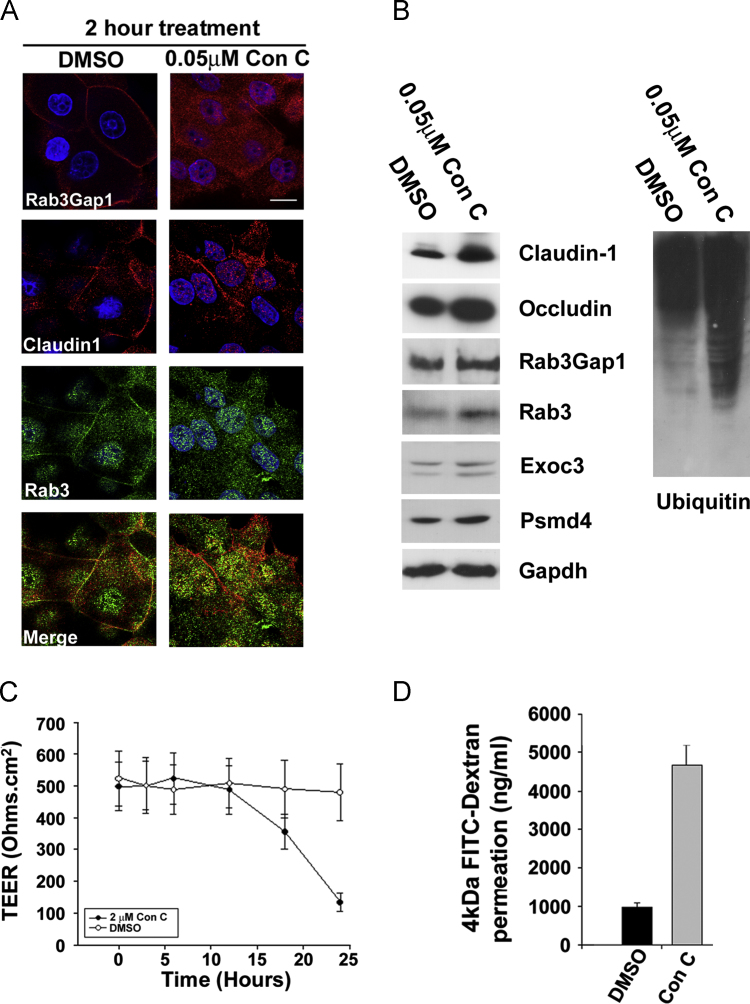
**Inhibition of exocytosis impairs paracellular barrier function, Claudin-1 expression and localisation.** (A) Confocal microscopy of Rab3Gap1, Claudin-1 and Rab3 in post-confluent REKs treated for 2 h with the exocytosis inhibitor Concanamycin C (Con C) or vehicle alone (DMSO). (B) Western blot analysis of Claudin-1, Rab3 and Rab3Gap1, Exoc3, Psmd4 and ubiquitin in keratinocytes treated with Concanamycin C for 2 h. Gapdh is the loading control. (C) Changes in Transepidermal electrical resistance (TEER) in response to Concanamycin C treatment for 24 h. (D) FITC dextran penetration in cells treated with Concanamycin C for 24 h. Error bars in C and D are s.d. for triplicate experiments representative of *n*=6. Bar 10 µm (A),

To determine whether inhibition of exocytosis was required to increase Claudin-1 expression and prevent cell surface localisation, we treated cells with the toxin Concanamycin C, which inhibits exocytosis by inhibiting vacuolar ATPase (Kataoka et al., 1994). Concanamycin C treatment for 2 h prevented Rab3Gap1 plasma membrane expression. Furthermore Claudin-1 was cytoplasmic and co-localised with Rab3 in the nucleus (Fig. 5A). By western blot, treated cells had increased levels of Claudin-1 and Occludin, and Exoc3 while levels of Rab3Gap1 and Exoc3 were unchanged (Fig. 5B). There was increased expression of Psmd4 and an increase in ubiquitinated protein species overall, suggesting that a decrease in proteasome-mediated degradation was unlikely to be involved in the accumulation of cytoplasmic Claudin-1 (Fig. 5B). Paracellular barrier function as determined by Transepithelial electrical resistance (TEER) and FITC-dextran penetration was reduced by concanamycin C treatment over a 24 h period (Fig. 5C and D). Therefore we show that inhibition of exocytosis is necessary and sufficient for the increased expression and cytoplasmic localisation of Claudin-1, and the disruption of barrier function.

**Fig. 6:**
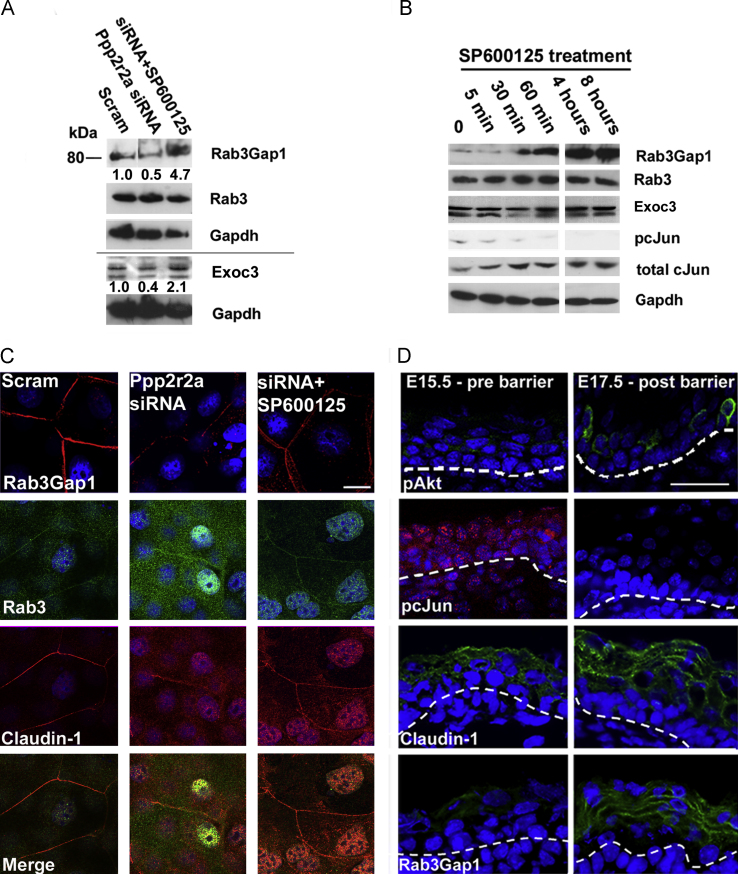
**Jun Kinase inhibition restores Rab3Gap1 expression and Claudin-1 cell surface expression. Cell surface Rab3Gap1 correlates with barrier acquisition during development.** A. Western blot analysis of Rab3Gap1 and Rab3 and Exoc3 in response to SP600125 treatment in Ppp2r2a kd cells. Numbers denote fold change in intensity compared to Scrambled controls after normalisation by levels of Gapdh, the loading control. (B) Western blot of Rab3Gap1, Rab3 and Exoc3 in REKs treated with SP600125 over an 8 h period. Note the reduction in phosphorylated cJun (pcJun) from 30 min correlates with the induction of Rab3Gap1 C. Immunofluorescence of Rab3Gap1 and Co-immunofluorescence of Claudin-1 and Rab3 in confluent scrambled, Ppp2r2a kd cells and Ppp2r2a kd cells treated with SP600125. D. Immunofluorescence of pcJun, pSerAkt, Ppp2r2a, Claudin-1 and Rab3Gap1 in E15.5 (Pre-barrier) and E17.5 (Post barrier) mouse epidermis, showing cell surface localisation of both Rab3Gap1 and Claudin-1 post barrier acquisition. Dotted line indicates the dermal epidermal boundary. Bar 10 µm (C) and 50 µm (D).

By Western blot we confirmed the reduction of Rab3Gap1 and Exoc3 expression in Ppp2r2a kd cells and restored expression in response to SP600125 treatment (Fig. 6A). Rab3Gap1 expression was increased within 30 min of SP600125 treatment, whereas Exoc3 expression increased only slightly after 8 h of SP600125 treatment. This correlated with loss of cJun phosphorylation (Fig. 6B). The predominant Rab3Gap1 protein in rat epidermal keratinocytes was the short (80kd) isoform that is transcribed from an alternate start site. ENCODE ChIP data from the UCSC genome browser showed this DNAse hypersensitive region was pulled down by cJun (Rosenbloom et al., 2012) We found 2 AP-1 (cJun) binding sites that were conserved in Human, Rat and Mouse in close proximity to the TATA box, suggesting that cJun binding could interfere with basal transcription from this site (Supplementary Fig. S4). Loss of Rab3Gap1 in Ppp2r2a kd confluent rat epidermal keratinocytes correlated with loss of cell surface co-localisation of Rab3 and Claudin-1. Cell surface expression of Rab3, Claudin-1 and Rab3Gap1 was restored with SP600125 treatment. (Fig. 6C), strongly suggesting that Rab3-mediated exocytosis was required for Claudin-1 transport and tight junction-related barrier function.

### Epidermal barrier acquisition correlated with cell surface Claudin-1 and Rab3Gap1 expression

Barrier acquisition correlates with a transient Akt activation and cJun dephosphorlyation during late mouse embryogenesis (O'Shaughnessy et al., 2009). Using these as proxies for barrier acquisition, we examined the expression of Claudin-1 and Rab3Gap1 *in vivo* in E15.5 and E17.5 mouse embryos, as this is the time period encompassing the barrier acquisition period (Fig. 6D). Claudin-1 expression was cytoplasmic pre-barrier, while expression was cell-surface in post barrier epidermis. Rab3Gap1 expression was increased and it too was on the cell surface. Therefore Rab3Gap1 cell surface localisation correlated with barrier acquisition *in vivo*.

### Rab3Gap1 is necessary for cell surface expression of Claudin-1 and Occludin and stratification

**Fig. 7:**
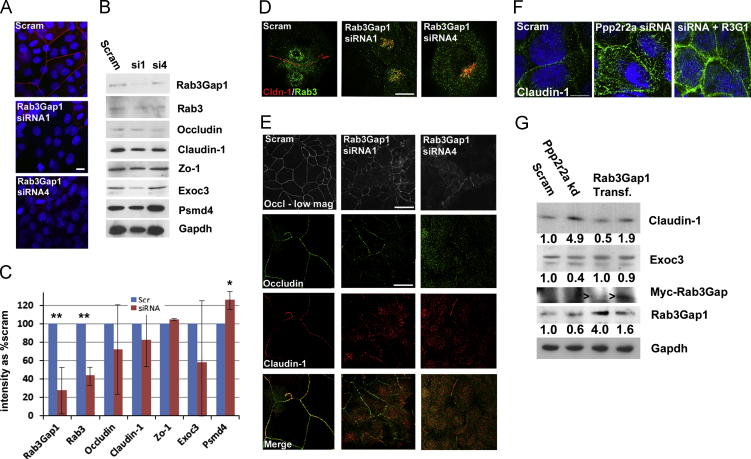
**Knockdown of Rab3Gap1 prevents cell surface localisation of Claudin-1 and Occludin. Rab3Gap1 rescues Claudin-1 localisation and expression in Ppp2r2a kd cells. (**A) Immunofluoresence of Rab3Gap1 in Scrambled controls and in two shRNA knockdowns of Rab3Gap1 (siRNA1 and siRNA4). (B) Western blot of Rab3Gap1 kd cells of Rab3Gap1, Claudin-1, Rab3a, Occludin, Zo-1, Exoc3 and Psmd4. Gapdh is the loading control. (C) Graph showing the mean percentage intensity in both kd lines (siRNA) compared to Scrambled controls (Scr) after normalisation by Gapdh. Error bars SD, *, *p*<0.05 **,*p*<0.005 (2-sided Students T-Test). (D) co-immunofluorescence of Claudin-1 and Rab3, showing peri-nuclear co-localisation in post-confluent Rab3Gap1 kd cells. E. Immunofluorescent analysis of Rab3Gap1 in confluent scrambled and Rab3Gap1 kd confluent keratinocytes. F. Expression of Claudin-1 in confluent scrambled controls, Ppp2r2a kd cells and kd cells transiently transfected with Rab3Gap1 (representative of 2 separate transfections). G. Western blot of Scrambled, Ppp2r2a kd cells and kd cells transfected with a Myc-tagged Rab3Gap1 (arrowhead). Numbers denote fold change in intensity of Claudin-1, Exoc3 and Rab3Gap1 compared to Scrambled controls after normalisation by levels of Gapdh, the loading control Bar 10 µm (A, D, E, F), 50 µm (E (low mag view)).

To determine whether Rab3Gap1 was necessary for the localisation of Occludin and Claudin to the cell surface, we knocked down Rab3Gap1 expression in REKs by shRNA expressing plasmids (Rab3Gap1 kd; Fig. 7A and B). In two separate Rab3Gap1 kd lines (siRNA1 and 4), we achieved significant reduction of Rab3Gap1 expression (10% and 50% of scrambled levels for si1 and 4, respectively). Rab3 was also reduced in both lines (Fig. 7B and C), There were no significant changes either in tight junction protein expression or the expression of Exoc3 or Psmd4 (Fig. 7B and C). Claudin-1 accumulated in the peri-nuclear region of differentiated keratinocytes and partially co-localised with Rab3 (Fig. 7D). The cells did not form any kind of transepidermal electrical resistance or act as a FITC-dextran penetration barrier and organotypic cultures were unable to stratify (not shown). Consistent with this putative defect in paracellular barrier, in post-confluent cells Rab3Gap1 kd cells, Occludin expression was discontinuous and reduced at the plasma membrane of knockdown cells (Fig. 7E). Claudin-1 did not co-localise with Occludin in the knockdown cells (Fig. 7E). Transient transfection of Rab3Gap1 into Ppp2r2a kd cells restored cell surface localisation of Claudin-1 (Fig. 7F) and reduced Claudin-1 expression levels (Fig. 7G). Exoc3 expression was also restored to levels in scrambled cells (Fig. 7G). Therefore Rab3Gap1 is required for Claudin-1 cell surface expression and epidermal barrier function, and the loss of Rab3Gap1 is responsible for Claudin-1 trafficking defects in the Ppp2r2a kd cells.

## Discussion

Taken together, these data suggest that the barrier defect in Ppp2r2a siRNA expressing cultures is a defect in the formation of tight junctions, specifically in Rab3-mediated Claudin-1 trafficking to the plasma membrane. Jun Kinase inhibition specifically rescued paracellular barrier function and Claudin-1 plasma membrane localisation. Inhibition of exocytosis caused cytoplasmic accumulation of Claudin-1 and reduced paracellular barrier function. These data suggest that constant exocytosis of Claudin-1 is required for barrier function, and that constant turnover of surface Claudin-1 occurs, consistent with the concept that epidermal tight junctions are not static permeability clamps. Rather, constant cell turnover in the epidermis requires tight junction turnover and reformation whilst maintaining barrier function. Recent FRAP data on Claudin turnover suggests that this is the case even in simple epithelia (Sasaki et al., 2003; Yamazaki et al., 2011). Claudin-1 mutations have been observed in the rare disease NISCH syndrome (ichthyosis and neonatal sclerosing cholangitis); (Hadj-Rabia et al., 2004; Feldmeyer et al., 2006). However, the complete loss of expression of Claudin-1 observed in these cases only results in a mild barrier defect. Epidermal Claudin-6 overexpression leads to a permeability barrier acquisition delay (Enikanolaiye et al., 2010;Turksen and Troy, 2002). Taken together this suggests that the over-expression and mis-localisation of tight junction proteins is more detrimental to epidermal barrier function than the loss of these proteins.

There are reports of links between Jun Kinase and epithelial barrier function. *Campylobacter jejuni is a* gut bacterium that causes diarrhoea by disrupting tight junctions, which is associated with Jun Kinase activation and redistribution of Occludin from the cell surface to an intracellular region (Chen et al., 2006). JNK inhibition in mammary epithelial cells increases Transepithelial electrical resistance in a dose-dependent manner (Carrozzino et al., 2009). This is consistent with our data showing a change in Occludin and Claudin-1 distribution, increase in FITC-dextran penetration in Ppp2r2a kd cells treated with the Jun Kinase inhibitor SP600125, and rescued of the expression of genes involved in exocytosis, including Rab3Gap1 and Exoc3.

Rab3 regulates exocytosis in a range of tissues (Weber et al., 1994). However, Rab3 does not contribute to the establishment of Claudin-1 containing tight junctions in fibroblasts (Yamamoto et al., 2003). Keratinocytes, unlike simple epithelia or fibroblasts, are stratified, and although the organ as a whole can be regarded as somewhat polarised, on a single cell level there is no clear evidence of polarisation in the upper epidermis (Helfrich et al., 2007; Kirschner et al., 2011). Rab3 may have a more widespread domain of exocytosis in keratinocytes and, as suggested by our *in vitro* data, is important for plasma membrane transport of Claudin-1 in keratinocytes. The exocyst, a protein complex required for targeted exocytosis, and Rab3 functionally interact in the fast, calcium dependent, exocytosis of neurotransmitters (Sudhof, 2004). As epidermal terminal differentiation and the initial formation of cell–cell contacts in keratinocytes is also calcium dependent, it is possible that the calcium gradient in the epidermis elicits the rapid exocytosis of tight junction components. In support of this is that siRNA knockdown of the calcium transporter ATP2C1 gene, mutated in the inherited skin blistering disease Hailey-Hailey disease, causes an increase in the expression of Claudins 1 and 4, and loss of cell-surface expression of tight junction proteins (Raiko et al., 2012).

Recently a role for microtubules in epidermal barrier function has been elucidated (Sumigray et al., 2012). Myosin IIA/B are required for bundling of the actin cytoskeleton and the non-centrosomal microtubule network in keratinocytes. Myosin IIA/B knockout mice cannot transport Occludin properly to tight junctions, leading to impairment of epidermal barrier function. Vesicular Rabs such as Rab3 associate with microtubule motors for transport (Horgan and McCaffrey, 2011). Therefore our data that Rab3Gap1 is required for correct Rab3 mediated Claudin-1 trafficking and Occludin localisation is consistent with a requirement for a microtubule network for barrier function.

Rab3Gap1 mutations cause Warberg microsyndrome, a disease characterised by microcephaly, microphthalmia, microcornia, cataracts, optic atrophy, cortical dysplasia, mental retardation, spastic diplegia, and hypogonadism (Morris-Rosendahl et al., 2010;Aligianis et al., 2005). No skin defects are reported suggesting that the effect of these mutations in the epidermis may be less severe that those we would predict from Rab3Gap1 loss, namely widespread epithelial barrier disruption. We propose that Rab3Gap1 controls exocytosis of a Claudin-1 pool, causing rapid acquisition of barrier function during development. Further investigation into drugs, like SP600125, that can restore the exocytosis of tight junction components, as well as the mechanisms of rapid barrier acquisition during development, could lead to therapies to restore barrier function not only in epidermis, but also other epithelia in premature infants.
