# Supplementary material for: Rab3Gap1 mediates exocytosis of Claudin-1 and tight junction formation during epidermal barrier acquisition
Source: Dev Biol. 2013 Aug 15;380(2):274–85. doi: 10.1016/j.ydbio.2013.04.034 (PMC3995087; doi:10.1016/j.ydbio.2013.04.034)

Supplementary information

Supplementary Figure S1

Table of fold change of differentially expressed genes in Ppp2r2a kd cells with a role in cell-cell junctions, on the right is a STRING analysis of interactions between these genes, up-regulated genes in red and down-regulated genes in green, unchanged genes, including Zo-1 (TJP1) and Occludin (OCLN) are grey.

Gene	Fold	Description
CLDN1	28	Claudin-1
FIIR	17	JAM-A
NPHP1	8.9	Nephrocystin-1
PARD6G	7.2	Partitioning defective 6 homolog gamma
CGNL1	0.35	Cingulin-like protein 1
SYMPK	0.31	Symplekin
ZYX	0.28	Zyxin
ACTN1	0.27	Alpha-actinin-1
HOMER1	0.27	Homer protein homolog 1
LASP1	0.27	LIM and SH3 domain protein 1
AFAP1	0.24	Actin filament-associated protein 1
ILK	0.23	Integrin-linked protein kinase
PTK2	0.22	Focal adhesion kinase 1
EZR	0.21	Ezrin
CLSTN1	0.19	Calsyntenin-1
CYFIP1	0.18	Cytoplasmic FMR1-interacting protein 1
FLNB	0.17	Filamin-B
CLIC4	0.14	Chloride intracellular channel protein 4
RDX	0.14	Radixin
VCL	0.13	Vinculin
MPP5	0.12	MAGUK p55 subfamily member 5
CALD1	0.11	Caldesmon
CTNNA1	0.09	Catenin alpha-1
MYH9	0.09	Myosin-9
SSX21P	0.09	Afadin- and alpha-actinin-binding protein
STAG1	0.08	Cohesin subunit SA-1
MSN	0.06	Moesin

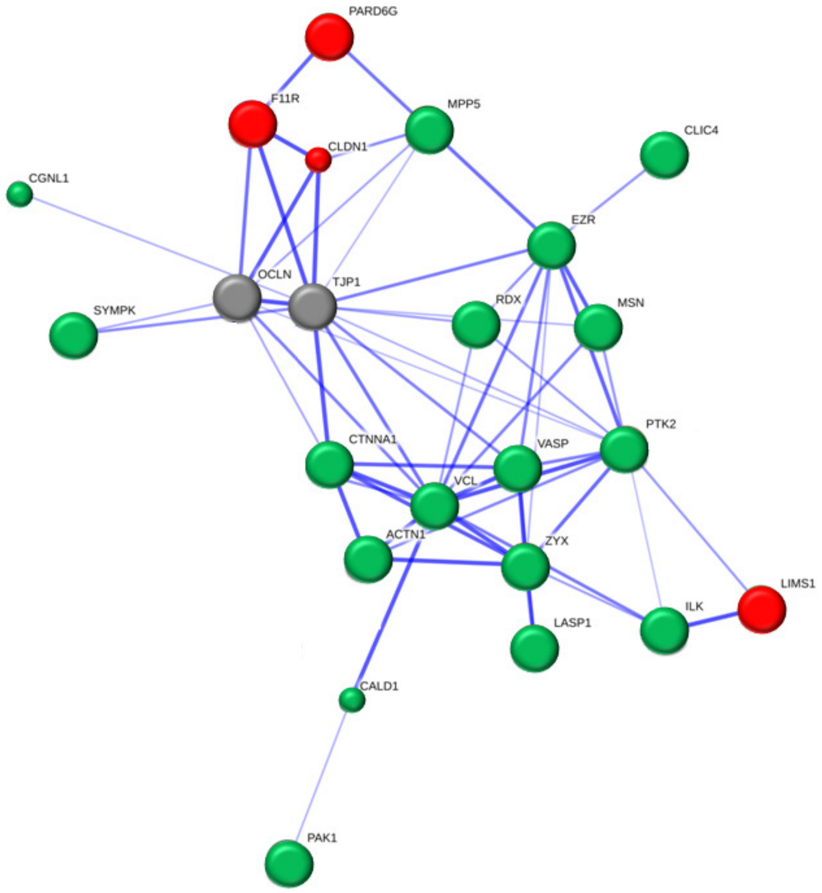

Supplementary Figure S2

Confocal ZX projections of Co-immunofluoresence of Claudin-1 and Occludin in post-confluent Scrambled, Ppp2r2a kd and Ppp2r2a kd cells treated with the Jun Kinase inhibitor SP600125. Bar 10 μm.

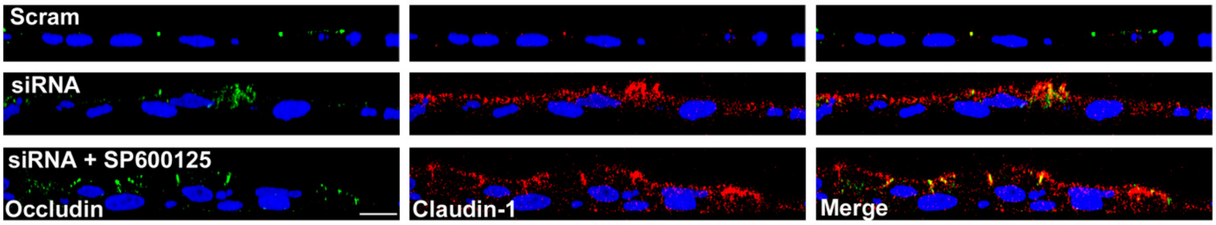

Supplementary Figure S3

Graphical representation of over-represented gene ontology groups in genes up- and down-regulated in Ppp2r2a kd cells, The blue line denotes the p<0.05 significance value.

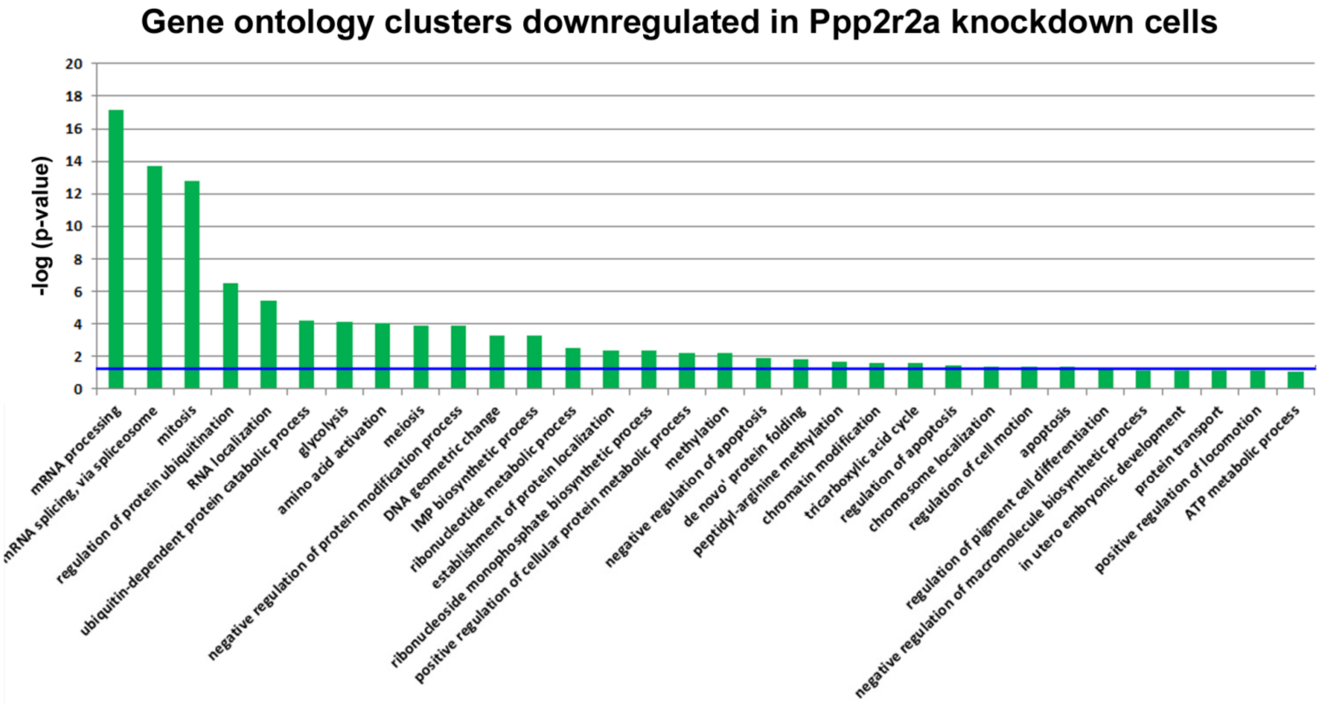

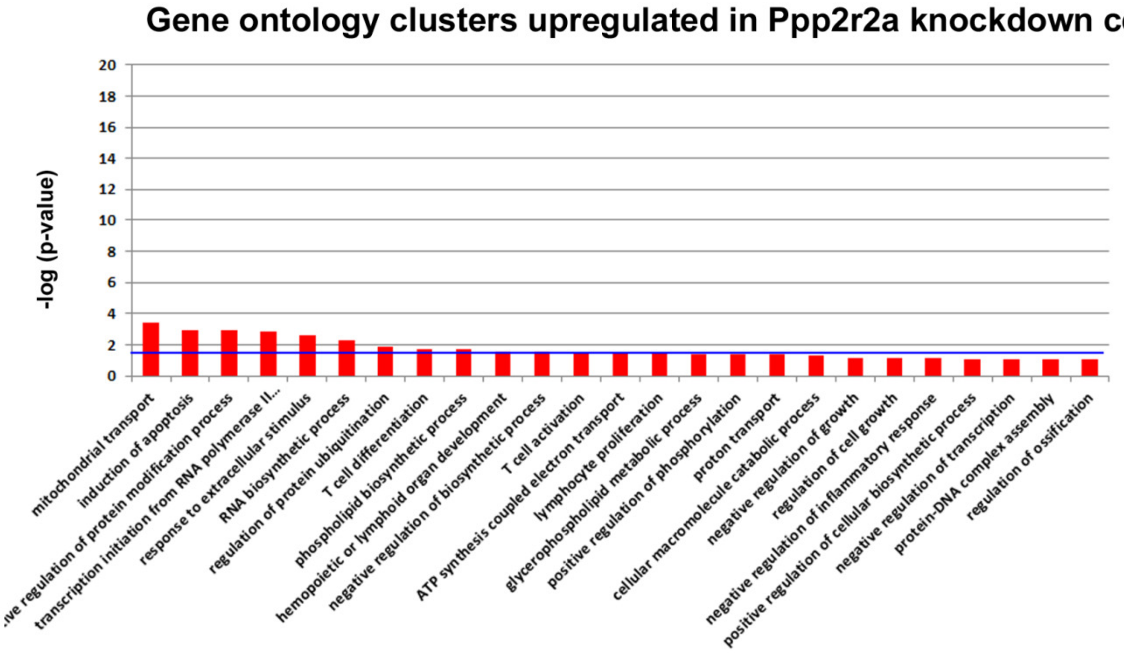

Supplementary figure S4

A. *In Silico* analysis of the alternative transcription start site for the Rab3Gap1 genes in humans, showing a DNaseI hypersensitivity site co-incident with ENCODE ChIP-seq data for cJun and JunD. B. Analysis of AP1 binding sites in this region showed conservation of 2 AP1 sites in close proximity to the TATA box in Human, Rat and Mouse.

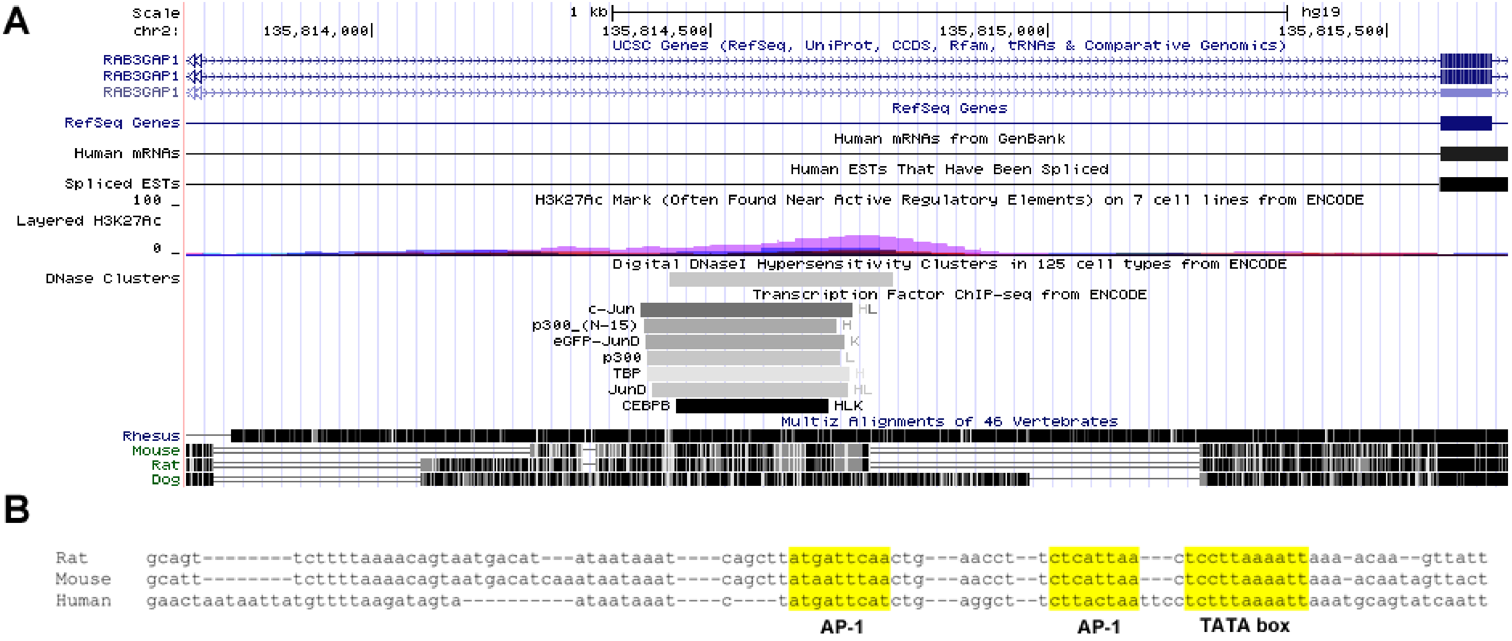

Supplement: Supplementary file 1 — Supplementary Material [file mmc1.pdf]
